# Supplementary material for: Chromothripsis during telomere crisis is independent of NHEJ, and consistent with a replicative origin
Source: Genome Res. 2019 May;29(5):737–49. doi: 10.1101/gr.240705.118 (PMC6499312; doi:10.1101/gr.240705.118)
Supplement: Supplemental Material [file supp_gr.240705.118_Supplemental_file_1.zip › contigs/annotated_contigs/DB85/contig.3.DB85_length_700_mean_cov_7.93571428571.docx]

**DB85_length_700_mean_cov_7.93571428571**

```
CGCCGCACACTGCCTCCACCAGTCACTCGATCCGGAAGATCCGACCCTACGTGATTCAGACTTGCTCAGCCCTTCTGACTTCAAAATCA
 >chr3:186944026-186944289 - E=2e-146
TCCTGGGTGAGTGAGTGAGGGGCAAGCTGACTCCCAAAGCCAGCTGTGGTTCTGGGAATGACCTACTGGCCCTTTCCAAGGCGTGTCTG

CTCACTCACTCGCTCACTCATTTGTCAGGGCAGGACACCTGGGATGGAGGTCCAGGAACCTGAGTCCAAGTGCAAGATATGATTC|G|T

GAGAAAATTCAAACAGCAGCAGCAGCCACCATTTTAACTACAAATATCAATGCAGCAGGCTACTCAAAGTTCTCTATGTGGTAGGACAA
>chr3:191777690-191777893 - E=2e-107
TGAGACTAATTAGTAAGAGGCTAATCTCCTGTTTCATCTTCACTAATTGATATTTCACTTTTGATCCTCCTTGCTCTGATTTGGGTAAT

ATCCACATAAAGAACCTGAATTTT|GTAAATATTT|TCTACTAAAAATACAAAAATTAGCTGGGTGTGGTGGCACATGCCTGTAGTCCC
                                   >chr16:90010874-90011097 + E=2e-122
AGCTACTTGGGAGGCTGAGGCAGAAGAATTGCTTGAACCCGGAAGGCGGAGGTTGCAGTGAGCCGAGATCGCACCTCTACACTCCAGCC

TGGTGAAAGAGCGAGACTCCGTCCCCCACCTGTCCCCACAAAAAAATTAGGAAGGTGTGGTGGCGTGTGTGTGTAGCCCCG
```
